# Time-dependent prognostic value of automated Ki67 assessment and its integration with molecular risk profiling in WHO grade 2 meningioma

**DOI:** 10.1186/s40478-026-02387-8

**Published:** 2026-07-27

**Authors:** Luisa Voßbeck, Felix Ehret, Eilis Perez, Helena Radbruch, Elisabeth G. Hain, Simone Schmid, Julia Onken, Merten Bohn, Nabiha Salman, Leonille Schweizer, David Capper, Moritz Armbrust, David Kaul

**Affiliations:** 1 Department of Radiation Oncology, Charité – Universitätsmedizin Berlin, Corporate Member of Freie Universität Berlin and Humboldt-Universität Zu Berlin, Berlin, Germany; 2 German Cancer Consortium (DKTK), Partner Site Berlin, A Partnership Between DKFZ and Charité – Universitätsmedizin Berlin, Berlin, Germany; 3 Department of Neuropathology, Charité – Universitätsmedizin Berlin, Corporate Member of Freie Universität Berlin and Humboldt-Universität Zu Berlin, Berlin, Germany; 4 Department of Neurosurgery, Charité – Universitätsmedizin Berlin, Corporate Member of Freie Universität Berlin and Humboldt-Universität Zu Berlin, Berlin, Germany; 5 Institute of Neurology (Edinger Institute), Goethe University Frankfurt, University Hospital, Frankfurt am Main, Germany; 6 Department of Radiation Oncology, Health and Medical University Potsdam, Olympischer Weg 1, 14471 Potsdam, Germany; 7 German Cancer Consortium (DKTK), Partner Site Frankfurt, German Cancer Research Center (DKFZ), Heidelberg, Germany; 8 Frankfurt Cancer Institute (FCI), Frankfurt am Main, Germany; 9 Goethe University Frankfurt, University Hospital, University Cancer Center (UCT), Frankfurt am Main, Germany

**Keywords:** Meningioma, Ki67, Digital pathology, QuPath, DNA methylation, Integrated risk score, WHO grade 2, Foundation model approach, Deep learning

## Abstract

WHO grade 2 meningiomas exhibit highly heterogeneous clinical courses. While the Ki67 proliferation index is a standard biomarker, its prognostic utility remains limited by methodological inconsistency and potential time-dependent dynamics. We evaluated an automated, artifact-adjusted Ki67 assessment and its integration with molecular risk profiling. 98 WHO grade 2 meningiomas (WHO 2021) were analyzed using an automated QuPath-based pipeline with HistoART for artifact exclusion. Molecular risk was defined by methylation and copy number profiling to calculate the integrated molecular-morphologic risk score by Maas et al. We employed extended Cox models to account for proportional hazards violations. Automated Ki67 values were significantly lower than routine pathological estimates (median 2.91% vs. 10%; *p* < 0.001) and correlated modestly with integrated risk scores (ρ = 0.26, *p* = 0.009). We identified a biphasic risk pattern: within the first 38 postoperative months, an automated Ki67 > 3.62% was a strong independent predictor for local recurrence (HR 5.06, *p* < 0.001) and progression-free survival (HR 4.15, *p* = 0.002), remaining significant alongside subtotal resection and the integrated risk group. Beyond 38 months, prognostic impact attenuated. Ki67 and the integrated molecular risk score contributed independently in multivariable models, suggesting complementary biological dimensions. Automated, artifact-adjusted Ki67 quantification provides time-dependent, independent prognostic information in WHO grade 2 meningioma, complementary to molecular risk stratification. It may serve as a cost-effective surveillance marker—both as an adjunct to molecular profiling and as a standalone tool where molecular testing is unavailable.

**Supplementary Information:**

The online version contains supplementary material available at 10.1186/s40478-026-02387-8.

## Introduction

Meningiomas are the most common primary intracranial tumors [1]. Among them, WHO grade 2 cases pose a particular clinical challenge due to their heterogeneous behavior, with recurrence rates ranging from 20 to 50% [2–7]. The clinical management of grade 2 meningiomas, especially regarding the use of adjuvant radiotherapy after gross total resection (GTR), remains an area of active investigation [5, 8, 9].

Recent advances in molecular profiling have demonstrated that DNA methylation-based classification and copy number variation (CNV) analysis can significantly improve risk stratification beyond histopathological grading [10–14]. The integrated molecular-morphologic risk score proposed by Maas et al. combines WHO grading, methylation family assignment, and specific chromosomal losses (1p, 6q, 14q) into a three-tiered classification that outperforms conventional grading in predicting recurrence [7, 12]. Our group has previously validated this classifier specifically for histologically confirmed grade 2 meningiomas, demonstrating that only about 50% of these tumors harbor the intermediate risk profile suggested by their histological grade, with potential implications for treatment decision-making [15]. However, comprehensive molecular profiling remains resource-intensive and is not universally available, highlighting a clinical need for accessible, cost-effective prognostic markers to guide decision-making [16].

The Ki67 proliferation index is one of the most widely available immunohistochemical markers in routine neuropathology. While higher Ki67 indices generally correlate with increasing WHO grade and progression-free and overall survival (PFS, OS), their prognostic utility in meningiomas remains debated [17–20]. This is partly driven by methodological inconsistencies due to varying pre-analytical staining protocols and a reliance on subjective visual estimation, resulting in poor standardization and high interobserver variability [21–24]. Digital pathology and automated whole-slide image (WSI) analysis, particularly via open-source platforms like QuPath, offer the potential for objective, high-throughput quantification [25]. Recent studies have suggested a prognostic value of automated Ki67 scoring in meningiomas, yet these approaches relied on manual tissue annotation [26, 27].

In the context of fully automated algorithms, the impact of tissue artifacts (e.g., tissue folds, air bubbles) has received limited attention [28]. To overcome this potential bias, deep learning-based artifact detection frameworks such as HistoART, which incorporate foundation model-based approaches (FMA), have recently emerged to automatically detect and exclude compromised regions [29]. However, the application of such advanced artifact-clearing pipelines to generate an artifact-adjusted Ki67 index remains unexplored in meningiomas.

Furthermore, the prognostic value of Ki67 may not be constant over time. Mirian et al. demonstrated that elevated Ki67 indices primarily drive early recurrences, resulting in a substantially shorter median time to relapse. While recurrence rates diverge sharply in the early postoperative phase, this discriminatory power diminishes over long-term follow-up [20]. Capturing this temporal dynamic is crucial for individualizing early surveillance strategies.

In this study, we addressed these limitations by implementing a quality-curated digital pathology pipeline. We utilized the HistoART framework to automatically exclude tissue artifacts, ensuring a high-quality, objective Ki67 assessment using QuPath, and investigated its time-dependent prognostic value and integration with molecular-morphologic risk scores. The primary aim of this work is to develop a standardized, reproducible proliferation metric that can be integrated with molecular risk stratification, rather than to re-evaluate visual assessment per se.

## Materials and methods

### Patient cohort and follow-up

In this single-center retrospective study, we included patients with histologically confirmed WHO grade 2 meningiomas treated surgically at Charité – Universitätsmedizin Berlin between 2007 and 2021 for primary and recurrent tumors. Inclusion criteria comprised available formalin-fixed, paraffin-embedded (FFPE) tumor tissue and clinical as well as radiographic follow-up of at least one year. All tumors were reassessed by an experienced board-certified neuropathologist to confirm grade 2 status according to the WHO 2021 Classification [7].

Demographic data, tumor characteristics (WHO grade, histological subtype, Ki67 index, location), and treatment modalities (Simpson grade, adjuvant radiotherapy) were extracted from medical records. Simpson grade was assessed using the surgery report and postoperative imaging report, with Simpson grades I–III defined as gross total resection (GTR) and Simpson grades IV–V defined as subtotal resection (STR) [16, 30]. Tumor location was classified as skull base or non-skull base. The integrated molecular-morphologic risk score was calculated using WHO grade, CNV (1p, 6q, 14q loss), and methylation family, as described by Maas et al. [12]. Tumors were dichotomized into skull base versus non-skull base location, consistent with available evidence that these groups differ in molecular driver mutations, resectability, and long-term recurrence patterns [31–33].

Clinical and radiographic follow-up intervals were calculated from the date of surgery to the last clinical contact and to the last available contrast-enhanced MRI, respectively. Local control (LC) was defined as the absence of local tumor recurrence or progression on contrast-enhanced MRI, assessed by a board-certified neuroradiologist. PFS was calculated from the date of surgery to local recurrence/progression or death of any cause. OS was defined as the interval from surgery to death of any cause. Censoring occurred at the last available imaging (LC) or last clinical contact (PFS, OS). PFS considers both radiographic recurrence/progression and death from any cause as events, whereas LC counts only radiographically confirmed recurrence. Deaths without documented recurrence, therefore, count as events for PFS but not for LC.

### Immunohistochemistry (IHC) and manual Ki67 quantification

Ki67 IHC was performed on 3 µm FFPE sections using a Benchmark XT Auto-Stainer (Ventana Medical Systems) with MIB1 antibody (M7240, Dako, 1:100) and CC1 buffer retrieval (pH 8.0; Ventana Medical Systems). Signal detection utilized the iVIEW DAB Kit (Ventana Medical Systems) with hematoxylin counterstaining. After automated dehydration, slides were coverslipped (Leica CV5030) and digitalized using a Leica Aperio GT 450 DX scanner (40 × magnification, 0.26 µm/pixel). WSIs were archived in SVS format. Routine Ki67 index assessments by board-certified neuropathologists were retrieved. For reported ranges, the maximum value was recorded (e.g., 3–5% → 5%). Non-numerical descriptions were excluded. The retrieved Ki67 indices originate exclusively from the historical diagnostic reports and were used as reported. This was a deliberate design choice: these routine indices represent real-world clinical practice, which is the comparator of interest for an automated method intended to improve standardization. No central re-review or re-quantification was therefore performed, as a research-grade re-read would not reflect actual practice but would instead address a separate, complementary methodological question (namely, the quality of an optimized visual assessment). Because Ki67 is not part of the WHO grading scheme and no gold-standard visual protocol exists, the heterogeneity among these reports likely reflects a lack of standardization rather than differences in individual reporting quality. Only two cases were reported in purely qualitative terms (“scattered individual positive nuclei” and “focally somewhat elevated”, no value stated); these were excluded from the visual comparison because such phrasing cannot, by definition, be assigned a defensible numerical index, and they do not represent a distinct clinicopathological subgroup.

### Automated Ki67 quantification

Automated Ki67 quantification was performed using QuPath (version 0.6.0) [25]. To minimize systematic bias from WSI artifacts, tissue quality control was conducted using the HistoART framework [29]. This FMA utilizes a pre-trained UNI model (ViT-L/16 architecture) to robustly detect artifacts [34, 35]. The pipeline involved WSI-tiling via QuPath Groovy scripts, followed by Python-based background pre-filtering and contrast normalization. The FMA evaluated each tile for six artifact categories (folds, blur, air bubbles, damage, marker traces, blood). Tiles with an artifact probability > 0.8 were excluded; downstream analyses were restricted to the resulting high-quality tissue annotations. The HistoART framework targets only these technical categories and does not specifically identify biological features. Inflammatory infiltrates, for example, are not excluded and remain part of the analyzed tissue.

To validate QuPath’s positive cell detection parameters, five trained observers manually counted cells in five representative areas (~ 32,300 µm^2^ each). Based on the calculated intraclass correlation coefficients (ICC) and Bland–Altman plots, the optimal QuPath configuration was defined as: optical density (OD) sum detection; 0.5 µm pixel size; 8 µm background radius; 0 µm median filter; 1.5 sigma; cell area 10–400 µm^2^; detection threshold 0.08; max. background intensity 2; and nucleus DAB OD mean threshold 0.25.

**Fig. 1:**
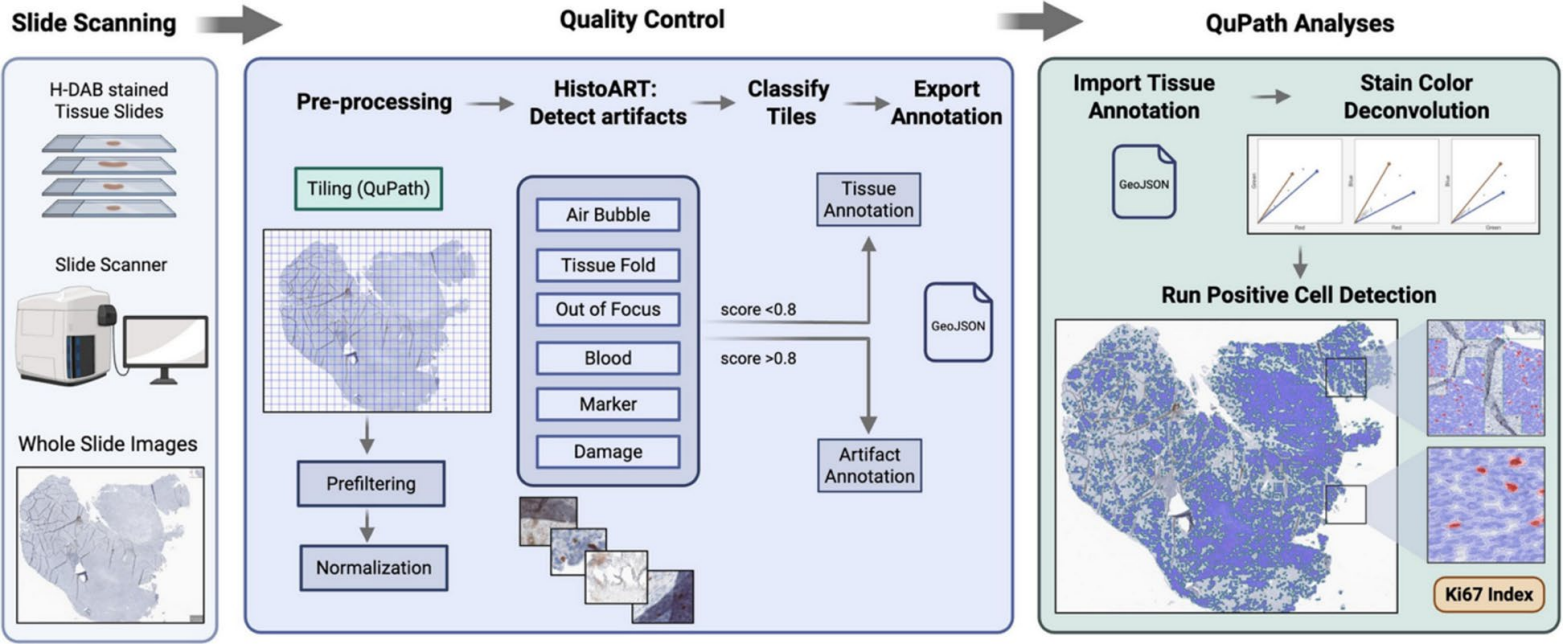
Digital pathology workflow for automated Ki67 assessment. Voßbeck, L. (Created in BioRender. (https://BioRender.com/l1k3wtb) is licensed under CC BY 4.0

For the full cohort, H-DAB optical density vectors were recalibrated on a representative region using QuPath's automated "estimate stain vectors" function. The validated cell detection parameters were applied via automated scripts to calculate the final automated, artifact-adjusted Ki67 index within FMA-cleared regions of each WSI (Fig. 1). The resulting value represents a global Ki67 index averaged across all FMA-cleared tissue. Focal elevations adjacent to infarction or necrosis are not selectively excluded.

As an exploratory analysis, each whole-slide image was additionally sampled with 75 tiles of  ≈ 0.25 mm^2^ (≈15 tiles in each of five sampling runs). The Ki67 index (percentage of Ki67-positive nuclei) was computed per tile, and an automated hotspot index was defined as the highest per-tile Ki67 index (restricted to tiles with ≥ 100 detected cells to ensure a sufficient denominator and avoid spurious maxima from sparsely populated tiles).

### Molecular analyses

DNA was isolated from FFPE tissue using the Maxwell RSC FFPE Plus Kit (AS1720, Promega). Following bisulfite conversion (EpiTect Fast, Qiagen) and restoration (Infinium HD FFPE DNA Restore), methylation profiling was performed on Illumina EPIC 850k arrays and imaged via an Illumina iScan system. Bioinformatic processing of raw IDATs and epigenetic classification were conducted using a locally deployed Brain Tumor Classifier (Frankfurt University Hospital) (v12.8) [10]. Sanger sequencing assessed TERT promoter mutations (C228, C250), while CNV profiles (R package *conumee* [36]) were used to identify homozygous CDKN2A/B deletions and chromosomal arm losses [37].

### Statistical analyses

Analyses and visualizations (violin plots, Kaplan–Meier curves) were performed using SPSS (v31.0) and RStudio (v2025.09.2; packages: *survival*, *survminer*, *survivalROC*, *ggplot2*). Continuous variables were assessed for normality via Shapiro–Wilk tests and reported as mean ± SD or median (IQR). Ki67 assessment reliability was evaluated using ICC (two-way mixed-effects, mean rating (k = 5), absolute agreement for inter-rater; two-way mixed-effects, single measures, consistency for inter-method) and Bland–Altman plots. Group comparisons utilized Chi-square, Fisher’s exact, Mann–Whitney U, or Kruskal–Wallis tests with Bonferroni-corrected post-hoc pairwise comparisons. Spearman’s rho was used for correlations. Median follow-up was calculated via the inverse Kaplan–Meier method.

Survival analyses for PFS, LC, and OS initially employed univariable Cox proportional hazards (PH) models and Harrell’s C-index. PH assumptions were verified using scaled Schoenfeld residuals [38]. Due to PH violations for Ki67, an extended Cox model with a time-interaction term (Ki67 × log(t + 1)) was implemented. To identify phase-specific effects, piecewise constant Cox models were constructed. The optimal time-split was determined via AIC-minimizing grid-search [39]. Within the resulting prognostic window, a time-dependent ROC analysis [40] with Youden index [41] established the optimal Ki67 cut-off. Finally, a multivariable extended Cox regression with AIC-based backward selection was performed, incorporating clinical covariates (age, sex, location, resection, radiotherapy, integrated risk group [12]). Multicollinearity was assessed via variance inflation factors (VIF). Significance was set at *p* < 0.05.

## Results

### Patient cohort and follow-up

Of the 121 screened cases, 23 were excluded during re-evaluation according to the 2021 WHO classification [7] due to insufficient mitotic counts (*n* = 22) or molecular upgrading (*n* = 1). The final cohort comprised 98 WHO grade 2 meningiomas (Table 1; 55.1% female; mean age 59.6 years). Histologically, the cohort was uniformly WHO grade 2, comprising atypical meningiomas graded by the WHO atypia criteria (93.9%) and chordoid meningiomas, a histological subtype designated WHO grade 2 (6.1%). Most surgeries (96.9%) were primary resections, with 66.3% of tumors located at the convexity/falx. GTR was achieved in 85.6% of documented cases. Adjuvant radiotherapy (15.3% overall) was administered significantly more frequently following STR (46.2%) than GTR (10.4%; *p* < 0.001). Over a median radiographic follow-up of 78.3 months, 37 recurrences and 21 deaths occurred. Local control rates at 1, 3, and 5 years were 93.9, 75.4, and 59.4%, respectively. Re-grading applied the full 2021 WHO criteria rather than mitotic count alone: within the retained cohort, WHO grade 2 was assigned on the basis of elevated mitoses (n = 80), histological soft features (n = 15), chordoid subtype (n = 6), and/or brain invasion (n = 6). The resulting reclassification rate of historical cases is consistent with the well-documented interobserver variability of meningioma grading [22] and the revised diagnostic thresholds introduced by the 2021 WHO criteria [7].

**Table 1:** Patient, tumor, and treatment characteristics of the study cohort (*n* = 98) with follow-up information

Characteristic	Mean	Range
Age at surgery (years)	59.6	21–86.9

	n	%
Sex: female/male	54/44	55.1/44.9
Primary / recurrent tumor	95/3	96.9/3.1
Tumor location: convexity or falx / skull base	65/31 *	66.3/31.6
WHO Grade 2 criterion: features of atypia / chordoid subtype	92/6	93.9/6.1
GTR (Simpson I–III) / STR (Simpson IV–V)	77/13 **	85.6/14.4 **
Adjuvant radiotherapy	15	15.3
Deaths	21	21.4
Recurrence/progression events	37	37.8

	Median	95% CI
Clinical follow-up (months)	82.3	71.4–93.3
Radiographic follow-up (months)	78.3	66.4–90.2

95% CI, 95% confidence intervals; GTR, gross total resection; STR, subtotal resection

^*^The remaining two cases were located in the posterior horn of the lateral ventricle

^**^Percentage of documented cases (n = 90)

### Molecular characterization

Methylation profiling and CNV analysis (Table 2) showed frequent losses of 22q (80.6%), 1p (59.2%), 14q (34.7%), and 6q (28.6%). Methylation families were benign (53.1%), intermediate (42.9%), and malignant (4.1%). The integrated risk score classified 30.6, 56.1, and 13.3% of tumors as low, intermediate, and high risk, respectively. The frequency of 1p, 6q, and 14q losses increased significantly across integrated risk groups (all *p* < 0.001), while 22q losses were distributed more evenly (*p* = 0.069). We observed a significant association between sex and risk group (*p* = 0.002): females were more likely to harbor low-risk tumors (44.4 vs. 13.6% in males), whereas high-risk tumors were more frequent in males (20.4 vs. 7.4% in females).

**Table 2:** Molecular-morphological characteristics of the WHO grade 2 study cohort (*n* = 98)

Characteristic	*n*	%
CNV: Chromosomal losses		
None	9	9.2
1p	58	59.2
6q	28	28.6
14q	34	34.7
22q	79	80.6
Methylation family		
Benign	52	53.1
Intermediate	42	42.9
Malignant	4	4.1
Integrated risk group		
Low	30	30.6
Intermediate	55	56.1
High	13	13.3

Methylation family according to the Brain Tumor Classifier v12.8 (Capper et al. [10])

Integrated risk group according to the Integrated Molecular-Morphologic Risk Score (Maas et al. [12])

### Ki67 quantification: automated versus visual assessment

Validation of QuPath’s cell detection parameters against a manual reference (five raters; inter-rater ICC = 0.989) demonstrated excellent agreement between the QuPath configuration and the raters’ mean Ki67 (ICC = 0.998), with a minimal mean absolute deviation of 0.239% (± SD 0.241) and negligible systematic bias (mean difference 0.08%; 95% LoA:  − 0.39–0.55%).

**Fig. 2:**
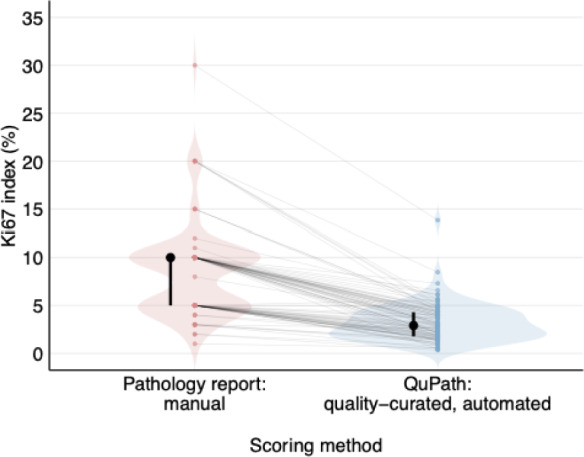
Intra-individual comparison of Ki67 index scoring methods. Violin plots illustrate the data distribution, with black indicators representing the median (dot) and interquartile range (line). Individual data points are displayed centrally; black lines connect corresponding measurements for the same patient, highlighting a systematic shift towards higher Ki67 indices in visual assessments compared with automated measurements

In the full cohort, retrospective pathology reports exhibited substantial reporting heterogeneity: 59.2% of cases lacked information on the assessed tumor region, and results were inconsistently provided as thresholds, focal values, or ranges. Inter-method reliability between these visual reports and automated, artifact-adjusted measurements was moderate (ICC = 0.534), indicating moderate inter-method reliability [42]. In the 96 cases with a documented visual index, visual assessments yielded significantly higher median Ki67 indices (10.0%, IQR 5–10%) than automated measurements (2.96%, IQR 1.78–4.30; *p* < 0.001) (Fig. 2). Bland–Altman analysis confirmed a pronounced proportional bias (mean difference 5.20%; 95% LoA: –1.63–12.02%), demonstrating low interchangeability. Consequently, the visual indices were excluded from further analyses.

As an exploratory analysis, we applied the survival models to the retrospective visual indices and additionally derived an automated, tile-based hotspot index. The automated hotspot (median 10.1%) was similar to the routine visual indices (median 10.0%; ρ = 0.63) and correlated with the global measure (ρ = 0.74). In early-phase models, both the hotspot and global indices were significant on dichotomization at their respective cut-offs, with comparable discrimination (AUC ≈ 0.66–0.70; Supplementary Tables S1 and S2).

### Association of Ki67 with molecular markers

**Fig. 3:**
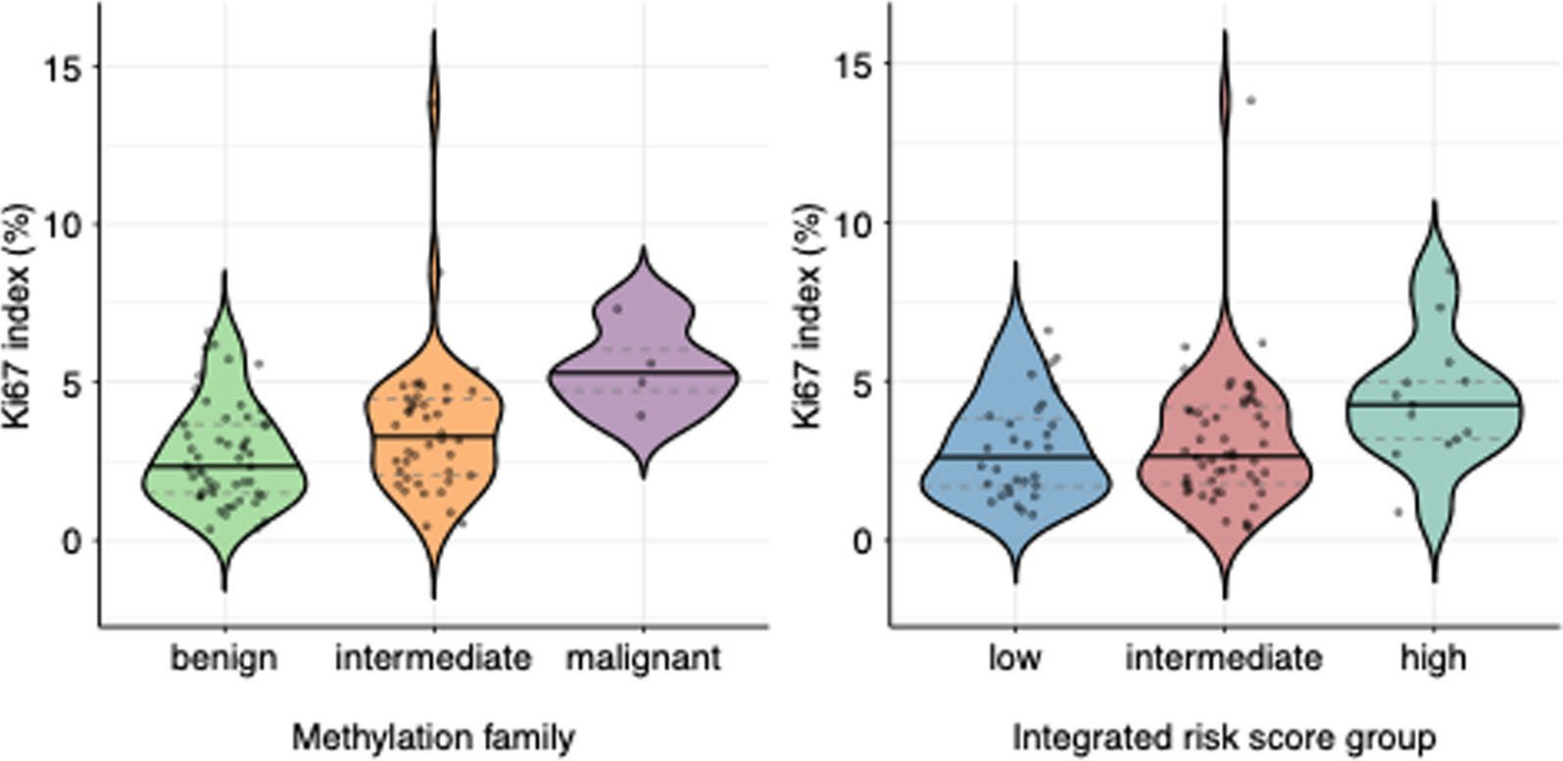
Automated Ki67 indices stratified by methylation family (left) and integrated risk score (right). Violin width indicates data density, with dots representing individual values. Black and gray lines denote the median and interquartile range (IQR), respectively. (Left) The distributions progressively shift toward higher Ki67 values from benign to intermediate and malignant methylation families according to the Brain Tumor Classifier v12.8 (Capper et al. [10]). (Right) While medians of the low and intermediate integrated risk groups (Maas et al. [12]) are similar, the intermediate risk group exhibits high-value outliers. Overall, Ki67 distributions are distinctly elevated in the high-risk group

Automated Ki67 indices increased progressively across benign (median 2.34%), intermediate (3.29%), and malignant (5.29%) methylation families (Fig. 3; Kruskal–Wallis *p* = 0.004; Spearman ρ = 0.30, *p* = 0.003; Table 3), with post-hoc significance between benign and malignant groups (*p* = 0.011). Among individual chromosomal losses, only 22q was significantly associated with higher median Ki67 (1.85 vs. 3.16%; *p* < 0.001), though the cumulative number of losses correlated positively with Ki67 (ρ = 0.27, *p* = 0.007). Ki67 indices also differed significantly across integrated risk groups (Kruskal–Wallis *p* = 0.030; Fig. 3; Table 3), with the high-risk group (median 4.27%) significantly exceeding both intermediate (2.64%) and low-risk groups (2.61%; both *p* < 0.05). Overall, Ki67 correlated significantly with the integrated risk score (ρ = 0.26, *p* = 0.009) and risk group (ρ = 0.20; *p* = 0.047).

**Table 3:** Automated Ki67 indices stratified by molecular markers

Molecular marker	Ki67 index (%): Median (IQR)		
Methylation family	Benign	Intermediate	Malignant
	2.34 (1.48–3.69)	3.29 (2.03–4.51)	5.29 (4.21–6.88)

CNV	No loss	Loss	
1p	2.29 (1.53–3.84)	3.18 (1.94–4.46)	
6q	2.85 (1.76–4.31)	3.10 (1.61–4.30)	
14q	2.55 (1.53–4.24)	3.28 (2.24–4.51)	
22q	1.85 (1.07–2.89)	3.16 (1.96–4.55)	

Risk group	Low	Intermediate	High
	2.61 (1.62–3.96)	2.64 (1.76–4.29)	4.27 (3.10–5.29)

IQR, interquartile range; methylation family according to Brain Tumor Classifier v12.8 (Capper et al. [10]); CNV, copy number variations; risk group according to integrated molecular-morphologic risk score (Maas et al. [12])

### Time-dependent prognostic effect of Ki67 in univariable Cox models

In univariable, standard Cox models assuming proportional hazards, the automated continuous Ki67 index showed no significant association with PFS (*p* = 0.414), LC (*p* = 0.516), or OS (*p* = 0.751). However, scaled Schoenfeld residuals revealed a significant violation of the PH assumption for PFS (*p* = 0.021) and OS (*p* < 0.001), with visual inspection indicating a similar pattern for LC (*p* = 0.360).

An extended Cox model with a time-varying interaction term (Ki67 × log(time + 1)) confirmed a significant baseline effect of the Ki67 index for PFS (HR 1.82; 95% CI 1.03–3.25; *p* = 0.049) and OS (HR 14.07; 95% CI 2.73–72.68; *p* = *0.*002), with significant attenuation over time for OS (interaction HR 0.51; *p* = 0.002) and a similar trend for PFS (HR 0.85; 95% CI 0.71–1.02; *p* = 0.072). No significant effects were observed for LC. Discriminatory performance was modest (C-index: 0.57–0.62).

To capture this temporal dynamic with a discrete time split, an AIC-minimizing grid search identified a convergent threshold at 38 months for both PFS (AIC 357.72) and LC (AIC 301.53; Supplementary Fig. S1), consistent with previously reported periods of maximal prognostic discrimination [20, 43]. Due to limited OS events (*n* = 21), further threshold determination was omitted for this endpoint.

In piecewise constant Cox models, the continuous Ki67 index significantly predicted PFS in the early phase (≤ 38 months; HR 1.24 per percentage point; 95% CI 1.07–1.42; *p* = 0.004), with a significant change in the late phase (interaction *p* = 0.003) and trend towards a risk reduction with increasing Ki67 indices (HR 0.75; 95% CI 0.56–1.00; *p* = 0.052). A similar pattern emerged for LC (interaction *p* = 0.033), with higher Ki67 indices showing a non-significant trend toward increased early-phase risk (HR 1.18; 95% CI 0.99–1.40; *p* = 0.060). Incorporating this time-split improved discriminatory performance over extended Cox models, yielding a C-index of 0.60 for both endpoints.

### Ki67 risk stratification

Time-dependent ROC analysis at 38 months identified an optimal Ki67 cut-off of 3.62% (AUC 0.66 for both endpoints, sensitivity 66.5%/69.7%; specificity 70.9%/72.0% for PFS/LC), stratifying patients into high (> 3.62%; n = 38) and low (≤ 3.62%; n = 60) proliferation groups. In piecewise constant Cox models, this dichotomization confirmed the time-dependent pattern (interaction *p* = 0.003 for PFS; *p* = 0*.*007 for LC) and further improved discriminatory performance (C-index: 0.61 for PFS; 0.63 for LC). In the early phase (≤ 38 months), Ki67 > 3.62% conferred a 3.4-fold increased PFS risk (HR 3.43; 95% CI 1.47–8.03; *p* = 0.004) and a 3.9-fold increased recurrence risk (HR 3.91; 95% CI 1.60–9.52; *p* = 0.003), while no significant association remained in the late phase (Table 4). The corresponding time-dependent ROC curves for both endpoints are provided in Supplementary Fig. S2.

**Table 4:** Univariable piecewise constant Cox models of Ki67 for local control and progression-free survival

Variables	LC		PFS	
	HR (95% CI)	*p*	HR (95% CI)	*p*
Continuous Ki67 index				
Early phase: Ki67 index (%)	1.18 (0.99–1.40)	0.060	1.24 (1.07–1.42)	0.004*
Late phase: Ki67 index (%)	0.74 (0.51–1.09)	0.132	0.75 (0.56–1.00)	0.052
Binary Ki67 risk group				
Early phase: Ki67 > 3.62% ^a^	3.91 (1.60–9.52)	0.003*	3.43 (1.47–8.03)	0.004*
Late phase: Ki67 > 3.62% ^a^	0.35 (0.08–1.56)	0.089	0.34 (0.10–1.18)	0.167

LC, local control; PFS, progression-free survival; HR, hazard ratio; 95% CI, 95% confidence intervals; *p*, *p*-value

Early phase: ≤ 38 months after surgery; late phase: > 38 months after surgery

^*^ *p* < 0.05 significant

^a^reference group: Ki67 ≤ 3.62%

**Fig. 4:**
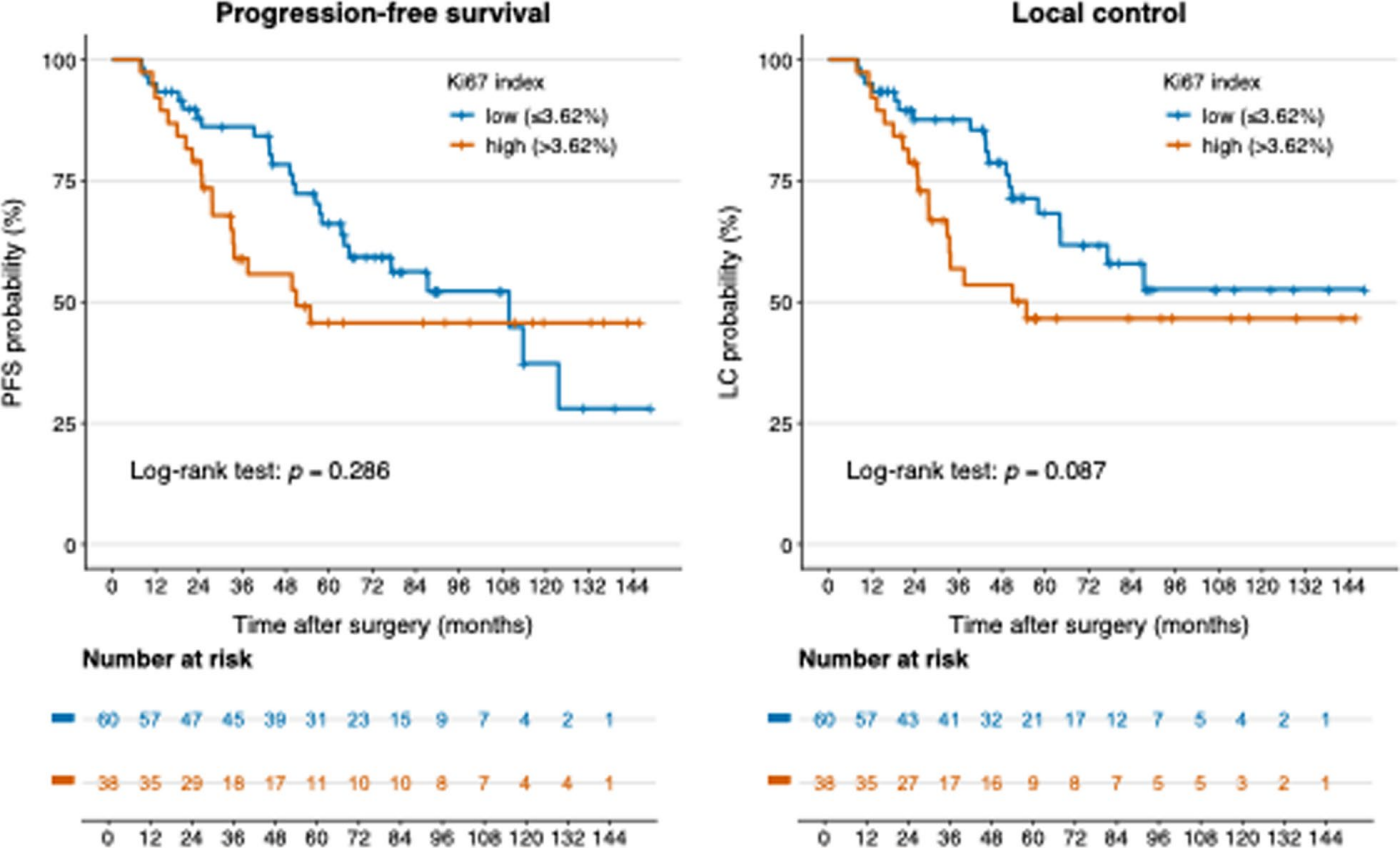
Kaplan–Meier estimates of progression-free survival and local control stratified by automated Ki67 groups. Blue lines represent patients with low Ki67 indices (≤ 3.62%), and orange lines represent high indices (> 3.62%). Both panels illustrate a pronounced early divergence of the survival curves up to approximately 38 months after surgery. This initial separation is followed by long-term convergence of the curves, resulting in non-significant global log-rank test results for both PFS (*p* = 0.286) and LC (*p* = 0.087). Vertical tick marks indicate censored observations, and number-at-risk tables for both cohorts are displayed beneath the x-axis

Kaplan–Meier analyses corroborated this temporal dynamic (Fig. 4). Although global log-rank tests did not reach significance due to late-stage curve convergence (PFS: *p* = 0.286; LC: *p* = 0.087), early separation was prominent. Within 38 months, PFS events occurred in 42.1% of high-Ki67 versus 13.3% of low-Ki67 patients, with substantially shorter median PFS (50.9 vs. 109.8 months) and time to recurrence (54.8 months vs. not reached) in the high-Ki67 group. Consistently, early recurrence was associated with significantly higher Ki67 indices (3.95 vs. 2.51%; *p* = 0.022), whereas no difference was observed over the entire follow-up (*p* = 0.429). In a sensitivity analysis restricted to primary resections (n = 95), the early-phase effect of a Ki67 > 3.62% remained essentially unchanged (LC HR 3.77, 95% CI 1.53–9.26, *p* = 0.004; PFS HR 3.30, 95% CI 1.40–7.80, *p* = 0.006; Supplementary Table S3), indicating that the inclusion of the few recurrent cases did not confound the findings. Within the high-Ki67 subgroup (> 3.62%, n = 38), patients with an early event (≤ 38 months, n = 16) did not differ from the remainder in their Ki67 level (median 4.49 vs. 4.75%, *p* = 0.668) but showed a significantly higher molecular burden (1p loss 100 vs. 50%, *p* = 0.001; 6q loss 50 vs. 9%, *p* = 0.008; higher integrated risk group *p* = 0.013; Supplementary Table S4).

### Univariable Cox regression analysis of clinical and molecular variables

The proportional hazards assumption was met for all variables and endpoints. In univariable Cox regression, male sex (HR 2.46; 95% CI 1.26–4.79; *p* = 0.008) and STR (HR 2.98; 95% CI 1.53–5.82; *p* = 0.001) were significant negative prognostic factors for LC, with similar significant effects on PFS (male sex: HR 2.29; 95% CI 1.26–4.17; *p* = 0.007; STR: HR 2.96; 95% CI 1.60–5.48; *p* < 0.001). Older age (≥ 65 years) was associated with worse PFS (HR 2.16; 95% CI 1.20–3.89; *p* = 0.011) but not LC (*p* = 0.057). The integrated risk score was the strongest predictor of LC and PFS. Compared to low-risk patients, intermediate-risk (LC: HR 9.75; 95% CI 2.31–41.18; PFS: HR 5.48; 95% CI 1.93–15.57; both *p* ≤ 0.002) and high-risk groups (LC: HR 13.85; 95% CI 2.98–64.35; PFS: HR 7.94; 95% CI 2.48–25.46; both *p* < 0.001) showed markedly elevated risks. For OS, age ≥ 65 years (HR 8.03; 95% CI 2.69–23.95; *p* < 0.001) and STR (HR 3.11; 95% CI 1.20–8.10; *p* = 0.020) were the only significant predictors. The integrated risk score, tumor location, adjuvant radiotherapy, and sex did not significantly predict OS (all *p* > 0.05).

### Multivariable Cox regression analyses

For LC and PFS, extended multivariable Cox models with the 38-month time-split were fitted after confirming non-proportional hazards for Ki67 via Schoenfeld residuals and excluding multicollinearity (all VIF < 1.5). Following AIC-based backward selection, age, sex, and tumor location were excluded from the LC and PFS models.

The final models (Table 5) demonstrated excellent discrimination (C-index: 0.79 for LC, 0.76 for PFS). Early-phase Ki67 > 3.62% was independently associated with local recurrence (HR 5.06; 95% CI 2.00–12.78; *p* < 0.001) and PFS events (HR 4.15; 95% CI 1.72–10.04; *p* = 0.002), with no significant effect in the late phase. STR (HR 7.41/7.18 for LC/PFS), intermediate (HR 10.26/5.37 for LC/PFS), and high integrated risk groups (HR 7.14/4.19 for LC/PFS), and adjuvant radiotherapy (HR 0.21/0.26 for LC/PFS) emerged as additional independent predictors (all *p* < 0.01).

**Table 5:** Multivariable extended Cox analyses for local control (LC) and progression-free survival (PFS)

Test variable (vs. reference)	LC		PFS	
	HR (95% CI)	*p*	HR (95% CI)	*p*
Early phase: Ki67 > 3.62% (vs. ≤ 3.62%)	5.06 (2.00–12.78)	< 0.001*	4.15 (1.72–10.04)	0.002*
Late phase: > 3.62% (vs. ≤ 3.62%)	0.88 (0.18–4.23)	0.869	0.65 (0.18–2.38)	0.513
STR (vs. GTR)	7.41 (2.78–19.78)	< 0.001*	7.18 (2.76–18.72)	< 0.001*
Unknown resection status (vs. GTR)	1.20 (0.40–3.56)	0.742	1.51 (0.57–4.02)	0.407
Adjuvant RT (vs. no RT)	0.21 (0.06–0.70)	0.012*	0.26 (0.08–0.78)	0.002*
Intermediate integrated risk group (vs. low)	10.26 (2.38–44.25)	0.002*	5.37 (1.86–15.52)	0.002*
High integrated risk group (vs. low)	7.14 (1.47–34.61)	< 0.001*	4.19 (1.24–14.14)	0.021*

Early postoperative phase: ≤ 38 months, late phase: > 38 months

LC, local control; PFS, progression-free survival; HR, hazard ratio; 95% CI, 95% confidence intervals; *p*, *p*-value; STR, subtotal resection; GTR, gross total resection; RT, radiotherapy

Risk group according to the Integrated Molecular-Morphologic Risk Score (Maas et al. [12])

^*^ *p *< 0.05 significant

Given the low OS event rate and PH violations, continuous Ki67 was modeled with a time interaction term. Age ≥ 65 years (HR 11.67; 95% CI 3.30–41.30; *p* < 0.001) and STR (HR 3.56; 95% CI 1.23–10.33; *p* = 0.019) were the leading independent risk factors, whereas unknown resection status was non-significant (*p* = 0.637). Ki67 index exhibited an initially high mortality risk (baseline HR 16.36; 95% CI 2.34–114.47; *p* = 0.005) that rapidly declined over time (interaction HR 0.48; 95% CI 0.30–0.78; *p* = 0.003). Backward selection eliminated sex, tumor location, radiotherapy, and integrated risk score from the final model (C-index: 0.85; all VIF < 1.5).

## Discussion

This study presents three principal findings regarding Ki67 assessment in WHO grade 2 meningiomas: (1) automated, artifact-adjusted Ki67 quantification provides lower overall values compared to visual pathologist assessments; (2) the Ki67 index modestly correlates with molecular risk markers, including the integrated molecular-morphologic risk score [12]; and (3) the prognostic effect of Ki67 is time-dependent, driving early recurrence within the first 38 months, with attenuation of this effect thereafter during the long-term follow-up.

### Methodological considerations: automated versus visual Ki67

The significant discrepancy between visual assessments (median 10.0%) and automated measurements (median 2.91%) aligns with recent digital pathology findings [26, 44, 45]. While our visual median corresponds to reported ranges for WHO grade 2 meningiomas [3, 19, 20, 46–48], direct comparability is often limited by inconsistent documentation of focal versus global methodologies in retrospective reports. Furthermore, visual estimation ("eyeballing") is known to inherently produce higher indices than rigorous counting [49]. A likely contributor to this offset is the conventional practice of focusing assessments on hotspots of proliferative activity, even when not explicitly documented. In contrast, our automated pipeline evaluates the entire available tissue section globally. This methodological asymmetry is consistent with the finding by Thirunavu et al. that manual routine pathology indices most closely resemble automated maximum hotspot values rather than global means [26]. Importantly, this critique concerns the standardization and reproducibility of routine visual reporting rather than the underlying biology: the established association between proliferation and recurrence is not in question, and our automated approach is intended to capture this relationship objectively and reproducibly rather than to revise it. Its added value therefore lies in standardization and automation, and in resolving two aspects that the visual-assessment literature has not formally established—the time-dependent (early-phase) nature of the Ki67 effect and its independence from molecular risk within the same cohort.

Our automated median of 2.91% (full cohort, n = 98) closely matches the QuPath-based findings of Thirunavu et al., who reported an average Ki67 index of 2.3% for WHO grade 2 meningiomas across the single most representative tissue block. Although they additionally investigated a multi-block Ki67 index, their single-block protocol, utilizing the most representative tissue block selected by an expert neuropathologist, provides the most appropriate baseline for our cohort, which similarly relies on single-block routine staining [26]. However, variability across platforms—such as Visiopharm or Virapath—underscores the sensitivity of automated metrics to specific software configurations [45, 50, 51]. This necessitates the preliminary threshold alignment we performed and highlights the broader need for standardized digital Ki67 protocols to ensure reproducibility [52].

Future digital Ki67 assessments should ideally integrate both global and automated hotspot analyses to fully capture the spatial heterogeneity of proliferative activity. Ugwuowo et al. demonstrated that low-grade meningiomas are often characterized by rare, dense clusters of Ki67-positive cells, whereas higher-grade lesions display more generalized, tissue-wide elevation—a spatial distinction that a single global metric may incompletely reflect [44]. Consistent with this, Thirunavu et al. showed that automated hotspot evaluations yield substantially higher indices than global means (averaging 6.8% in grade 2 tumors), yet recurrence-free survival was best predicted by the global automated mean which aligns with the approach we adopted [26]. Systematically capturing proliferative heterogeneity across multiple tissue blocks and assessment strategies therefore represents an important direction for future work, and the reliance on a single tissue block with global quantification constitutes a limitation of the present study. In our cohort, an automated hotspot index (the tile with the highest Ki67 index) closely matched the routine visual indices (median 10.1% vs. 10.0%), consistent with visual reporting reflecting proliferative hotspots, and showed early-phase prognostic discrimination comparable to the global measure (Supplementary Table S2). This indicates that hotspot assessment was not required to capture the prognostic signal and that the global quantification we adopted is sufficient, while additionally offering greater standardization, reproducibility, and robustness to single-tile noise.

Finally, implementing the HistoART framework [29] addresses a critical shortcoming of current digital workflows by automating artifact exclusion. This approach mitigates the reliance on labor-intensive manual annotations and substantially reduces the issues of artifact-prone pipelines identified in earlier research [26, 27, 44, 45, 53–55]. To benchmark the artifact-adjusted pipeline, we compared it with a global QuPath quantification of the same slides performed without HistoART artifact exclusion, using cutoff-independent metrics. The two indices were highly correlated and prognostically equivalent: at 38 months the time-dependent AUC was 0.66–0.67 for both, and, evaluated at each index’s own ROC-optimal cut-off, the early-phase hazard ratios (LC ≈ 3.9; PFS ≈ 3.4) and concordance indices (≈ 0.60–0.62) were comparable (Supplementary Table S5). Artifact removal therefore did not materially improve prognostic discrimination in this cohort. Its value is methodological, objectively excluding technical artifacts (folds, blur, air bubbles, tissue damage, marker traces, blood) that could otherwise be misclassified as positive or negative nuclei and thereby safeguarding measurement validity and reproducibility, especially for smaller or artifact-rich specimens.

### Time-dependent prognostic dynamics of Ki67

A key novelty and central finding of this study is the demonstration of a time-dependent prognostic impact for Ki67. In uni- and multivariable analyses, a Ki67 index > 3.62% conferred a substantially increased risk of local recurrence and shorter PFS in the early postoperative phase (≤ 38 months) but did not significantly affect long-term outcomes. Continuous Ki67 indices were also predictive for PFS events in the early phase in univariable models but did not reach significance for local recurrence. Similarly, this dynamic extended to OS. Modeled continuously with a time interaction term, Ki67 demonstrated an initially high mortality risk that rapidly waned over time in uni- and multivariable analyses.

Our data-driven cut-off of 3.62% for local control and PFS prediction aligns closely with the 3.42% threshold reported by Thirunavu et al. using a comparable automated QuPath approach [26]. In contrast, it is slightly lower than the visually derived 4.1% threshold proposed by Shukla et al. [56]. As both reference studies utilized pan-grade cohorts (WHO grade 1–3), their optimal thresholds are inevitably shifted downward by the high prevalence of low-proliferating grade 1 tumors. This underscores the necessity of establishing a tailored cut-off to accurately capture the distinct proliferation dynamics and nuanced recurrence risk specifically within the intermediate WHO grade 2 subset.

Our findings align with and expand upon evidence of temporal risk dynamics. Bruna et al. noted a significantly shorter time to recurrence for high-Ki67 tumors (12.6 vs. 35.2 months) [57]. Similarly, Mirian et al. demonstrated that Ki67 predominantly drives early risk, reporting a 1-year recurrence incidence of 19% (Ki67 > 4%) versus 3% (Ki67 ≤ 4%) and a drastically shorter median time to recurrence (0.6 vs. 4.8 years), with recurrence rates ultimately converging at 10 years [20]. While most previous literature relied on standard proportional hazards models, often reporting an overall long-term prognostic effect of Ki67 over extended follow-up periods [3, 46, 48], this overall significance may be driven by the disproportionate event rate in the early phase. Indeed, in some studies not explicitly testing for time-dependency, published PFS curves frequently exhibit a similar biphasic pattern, visually diverging during the first 40–50 postoperative months before gradually converging over long-term follow-up [48, 56, 58]. While our statistical models successfully formalize this visually apparent trend, the exact temporal cut-offs and risk thresholds require prospective validation. We emphasize that the 38-month threshold is a data-driven optimum identified by AIC minimization rather than a biologically predefined cut-off. For LC, it was the distinct AIC optimum, whereas for PFS two near-equivalent local optima emerged at 38 and 56 months (ΔAIC = 1.2), with 38 months selected as the split convergent across both endpoints. The robust finding is the strong early-phase effect, whereas the apparent late attenuation must be interpreted cautiously given the limited number of patients still at risk in the high-Ki67 group at later time points.

### Integration with molecular risk and clinical implications

We observed a significant but modest correlation (Spearman ρ = 0.26–0.30) between the automated Ki67 index and molecular markers (methylation family, cumulative CNV, and the integrated risk score [12]). While previous studies similarly reported elevated Ki67 indices within aggressive methylation groups [14, 59–61], our modest correlation coefficients suggest that Ki67 is not merely a surrogate for molecular high-risk features, but captures a complementary biological dimension. This is mechanistically supported by the lack of overlap between CpG sites associated with Ki67 expression and the hypermethylated loci defining molecular high-grade meningiomas [62]. Reflecting this epigenetic divergence, our multivariable analyses identified both the early-phase Ki67 index and the integrated molecular risk group as independent predictors for local control and PFS. Notably, for OS, the time-dependent, continuous Ki67 index retained its independent prognostic value for early mortality, whereas the integrated risk score was eliminated, though this finding should be interpreted cautiously given the limited number of OS events (*n* = 21). Overall, a comprehensive prognostic evaluation likely benefits from incorporating both modern molecular stratification and automated proliferation assessment.

### Clinical implications

Building on the complementary and independent contributions of Ki67 and molecular risk demonstrated in our multivariable models, automated Ki67 assessment may inform postoperative management across different levels of diagnostic resource availability. In centers where comprehensive molecular profiling is accessible, a Ki67 index > 3.62% could prompt intensified early surveillance (e.g., 3–6-monthly MRIs) and expedited molecular workup, while molecular characterization remains essential regardless of Ki67 level to capture long-term recurrence risk beyond the 38-month prognostic window.

In settings where molecular testing is unavailable or delayed, an elevated Ki67 index may serve as a standalone early warning marker, identifying patients who warrant closer follow-up based on proliferative activity. This dual utility—as an adjunct to molecular profiling and as an independent marker when molecular data are absent—positions automated Ki67 as a pragmatic and widely accessible component of risk stratification in WHO grade 2 meningioma. At the same time, we acknowledge that while Ki67 immunohistochemistry is available in virtually all pathology laboratories, routine whole-slide digital pathology infrastructure is not yet universally established; the approach presented here is therefore best viewed in the context of the ongoing, rapid expansion of digital pathology rather than as an immediately ubiquitous tool.

### Limitations and future directions

Several limitations must be acknowledged. First, the retrospective, single-center design (*n* = 98) inherently introduces sampling biases and limits statistical power in smaller subgroups, as reflected in the wide confidence intervals observed for some of our estimates. Notably, adjuvant treatment was not randomized. The strong prognostic effect of radiotherapy observed here cannot be interpreted causally due to substantial confounding by indication, as patients with subtotal resections received irradiation significantly more frequently. Furthermore, substantial censoring and the declining number at risk in the high-Ki67 group at later follow-up (38 of 38 at baseline, 16 at 38 months, and 8 at 72 months; Supplementary Table S6) limit the statistical power to characterize the late phase; the attenuation of the Ki67 effect beyond 38 months should therefore be regarded as hypothesis-generating rather than as evidence of a truly vanishing risk. In addition, because necrotic or hypocellular regions can morphologically resemble technical artifacts (e.g., cautery-induced changes), the artifact classifier may not always separate them cleanly from genuine artifacts; this distinction was not formally validated and may introduce some variability into the global index. Relatedly, the specific architectural patterns of the atypical meningiomas were not systematically cataloged, and within-tumor regional histological heterogeneity (e.g., in cellularity, inflammation, or fibrous content) was not separately modeled. As Ki67 was quantified globally across the available high-quality tissue, a region-resolved analysis of histological patterns was beyond the scope of this single-block study.

Second, while our automated QuPath pipeline utilizing the HistoART foundation model is rigorously standardized, cell detection parameters and the 3.62% cut-off are optimized for our institutional pre-analytical conditions. Because variations in tissue processing, staining protocols, and scanners across laboratories affect optical density, this pragmatic threshold may require local recalibration. Consequently, a critical future direction is the cross-institutional standardization of digital pathology workflows, incorporating robust stain normalization techniques. Further, the data-driven dichotomization of Ki67 values (3.62% cut-off) and the 38-month time split are pragmatic constructs that carry an inherent risk of overestimation and thus require rigorous validation in independent, prospective cohorts to ensure reproducibility [63].

A potential methodological concern is a domain shift, as the underlying UNI model [35] was primarily trained on H&E-stained datasets. While recent evidence suggests that H&E-pretrained foundation models may not fully generalize to IHC-specific diagnostic tasks requiring stain interpretation [64], this limitation is less relevant in the context of artifact detection: tissue artifacts such as folds, blur, and air bubbles are defined by structurally invariant, stain-agnostic characteristics. Our visual validation confirmed that the model robustly excluded compromised regions, ensuring the high reliability of the resulting artifact-adjusted Ki67 index. Notably, UNI 2, the successor model trained on both H&E and IHC data [35, 65], has since been released, which may further mitigate this concern in future applications.

Looking forward, future research should leverage foundation models not only for artifact control, as demonstrated herein, but to extract biological information directly from routine histology. Recent work has established the feasibility of histology-based molecular surrogates, ranging from models that classify broad CNS tumor categories from H&E images across external cohorts to emerging meningioma-specific frameworks inferring methylation subtypes and chromosomal losses from whole-slide images [66–69]. Critically, however, our data suggest that automated Ki67 captures a biologically distinct and complementary dimension: the modest correlation with the integrated molecular risk score and its independent prognostic contribution in multivariable models indicate that proliferative activity and epigenetic risk are not redundant. Furthermore, automated Ki67 specifically resolves the early, time-dependent recurrence risk within the first 38 postoperative months in this study—a temporal dynamic that epigenetic classifiers alone may not capture. Prospectively validated, multimodal integration of both approaches therefore may represent the next step toward precision risk stratification in WHO grade 2 meningioma.

## Conclusions

Automated, artifact-adjusted Ki67 quantification reveals a time-dependent prognostic effect in WHO grade 2 meningiomas, with independent predictive value for early recurrence within the first 38 postoperative months—a clinically critical window for surveillance decisions. The modest correlation with the integrated molecular risk score and the independent multivariable contributions of both markers indicate that proliferative activity and epigenetic risk represent complementary rather than redundant biological dimensions of tumor behavior. Importantly, given its independence from molecular risk, automated Ki67 may serve as a standalone early surveillance marker in settings where molecular profiling is unavailable, while retaining additive prognostic value when integrated into comprehensive molecular workup. These findings support the concept of a tiered, risk-adapted approach to postoperative management, in which standardized Ki67 assessment contributes meaningfully across the full spectrum of diagnostic resources. Prospective multicenter validation is needed to establish generalizable thresholds and confirm the clinical utility of this integrated framework.

## Supplementary Information

Additional file1 (DOCX 229 KB)

## Data Availability

The datasets generated and analyzed during the current study are available from the corresponding author on reasonable request.

## Abbreviations

AIC: Akaike information criterion
AUC: Area under the curve
CI: Confidence interval
CNV: Copy-number variation
DAB: 3,3’-Diaminobenzidine
FFPE: Formalin-fixed, paraffin-embedded
FMA: Foundation model-based approach
GTR: Gross total resection
H&E: Hematoxylin and eosin
HR: Hazard ratio
ICC: Intraclass correlation coefficient
IHC: Immunohistochemistry
IQR: Interquartile range
LC: Local control
LoA: Limits of agreement
MRI: Magnetic resonance imaging
OS: Overall survival
PFS: Progression-free survival
PH: Proportional hazards
ROC: Receiver operating characteristic
RT: Radiotherapy
STR: Subtotal resection
VIF: Variance inflation factor
WHO: World Health Organization
WSI: Whole-slide image

## References

[1] Ostrom QT, Price M, Neff C, Cioffi G, Waite KA, Kruchko C, et al (2023) CBTRUS statistical report: primary brain and other central nervous system tumors diagnosed in the United States in 2016–2020. Neuro-Oncol 25:iv1–99. 10.1093/neuonc/noad149 10.1093/neuonc/noad149 PMC10550277 37793125

[2] Champeaux C, Houston D, Dunn L (2017) Atypical meningioma. A study on recurrence and disease-specific survival. Neurochirurgie (Paris) 63:273–281. 10.1016/j.neuchi.2017.03.004 10.1016/j.neuchi.2017.03.004 28882609

[3] Barrett OC, Hackney JR, McDonald AM, Willey CD, Bredel M, Fiveash JB (2019) Pathologic predictors of local recurrence in atypical meningiomas following gross total resection. Int J Radiat Oncol Biol Phys 103:453–459. 10.1016/j.ijrobp.2018.09.019 30253235 10.1016/j.ijrobp.2018.09.019

[4] Nadeem M, Goyal-Honavar A, Sravya P, Beniwal M, Santosh V, Dwarakanath S (2024) Prognostic factors and outcomes in World Health Organization grade 1 and grade 2 intracranial meningiomas-5-year institutional experience. World Neurosurg 187:e331–e339. 10.1016/j.wneu.2024.04.082 38649022 10.1016/j.wneu.2024.04.082

[5] Goldbrunner R, Stavrinou P, Jenkinson MD, Sahm F, Mawrin C, Weber DC et al (2021) EANO guideline on the diagnosis and management of meningiomas. Neuro Oncol 23:1821–1834. 10.1093/neuonc/noab150 34181733 10.1093/neuonc/noab150 PMC8563316

[6] Millward CP, Keshwara S, Islim AI, Zakaria R, Jenkinson MD (2023) Clinical presentation and prognosis. In: Zadeh G, Goldbrunner R, Krischek B, Nassiri F (eds) Biol Clin Landsc Meningiomas. Springer International Publishing, Cham, pp 5–20 10.1007/978-3-031-29750-2_2 37432616

[7] Sahm F, Perry A, von Deimling A, Claus EB, Mawrin C, Brastianos PK et al (2021) Meningiomas, 5th ed. International Agency for Research on Cancer, Lyon, pp 283–298

[8] Wujanto C, Chan TY, Soon YY, Vellayappan B (2022) Should adjuvant radiotherapy be used in atypical meningioma (WHO grade 2) following gross total resection? a systematic review and meta-analysis. Acta Oncol 61:1075–1083. 10.1080/0284186X.2022.2116994 36052871 10.1080/0284186X.2022.2116994

[9] Lee G, Lamba N, Niemierko A, Kim DW, Chapman PH, Loeffler JS et al (2021) Adjuvant radiation therapy versus surveillance after surgical resection of atypical meningiomas. Int J Radiat Oncol Biol Phys 109:252–266. 10.1016/j.ijrobp.2020.08.015 32777336 10.1016/j.ijrobp.2020.08.015

[10] Capper D, Jones DTW, Sill M, Hovestadt V, Schrimpf D, Sturm D et al (2018) DNA methylation-based classification of central nervous system tumours. Nature 555:469–474. 10.1038/nature26000 29539639 10.1038/nature26000 PMC6093218

[11] Sahm F, Schrimpf D, Stichel D, Jones DTW, Hielscher T, Schefzyk S et al (2017) DNA methylation-based classification and grading system for meningioma: a multicentre, retrospective analysis. Lancet Oncol 18:682–694. 10.1016/S1470-2045(17)30155-9 28314689 10.1016/S1470-2045(17)30155-9

[12] Maas SLN, Stichel D, Hielscher T, Sievers P, Berghoff AS, Schrimpf D et al (2021) Integrated molecular-morphologic meningioma classification: a multicenter retrospective analysis, retrospectively and prospectively validated. J Clin Oncol 39:3839–3852. 10.1200/JCO.21.00784 34618539 10.1200/JCO.21.00784 PMC8713596

[13] Nassiri F, Mamatjan Y, Suppiah S, Badhiwala JH, Mansouri S, Karimi S et al (2019) DNA methylation profiling to predict recurrence risk in meningioma: development and validation of a nomogram to optimize clinical management. Neuro-oncol 21:901–910. 10.1093/neuonc/noz061 31158293 10.1093/neuonc/noz061 PMC6620635

[14] Choudhury A, Magill ST, Eaton CD, Prager BC, Chen WC, Cady MA et al (2022) Meningioma DNA methylation groups identify biological drivers and therapeutic vulnerabilities. Nat Genet 54:649–659. 10.1038/s41588-022-01061-8 35534562 10.1038/s41588-022-01061-8 PMC9374001

[15] Ehret F, Perez E, Teichmann D, Meier S, Geiler C, Zeus C et al (2024) Clinical implications of DNA methylation-based integrated classification of histologically defined grade 2 meningiomas. Acta Neuropathol Commun 12:74. 10.1186/s40478-024-01739-6 38720399 10.1186/s40478-024-01739-6 PMC11080225

[16] Wang JZ, Landry AP, Raleigh DR, Sahm F, Walsh KM, Goldbrunner R et al (2024) Meningioma: international consortium on meningiomas consensus review on scientific advances and treatment paradigms for clinicians, researchers, and patients. Neuro-oncol 26:1742–1780. 10.1093/neuonc/noae082 38695575 10.1093/neuonc/noae082 PMC11449035

[17] Liu N, Song S-Y, Jiang J-B, Wang T-J, Yan C-X (2020) The prognostic role of Ki-67/MIB-1 in meningioma: a systematic review with meta-analysis. Med (Baltimore) 99:e18644. 10.1097/MD.0000000000018644 10.1097/MD.0000000000018644 PMC7478528 32118704

[18] Aung TM, Ngamjarus C, Proungvitaya T, Saengboonmee C, Proungvitaya S (2024) Biomarkers for prognosis of meningioma patients: a systematic review and meta-analysis. PLoS ONE 19:e0303337. 10.1371/journal.pone.0303337 38758750 10.1371/journal.pone.0303337 PMC11101050

[19] Broechner A, Maier AD, Mirian C, Sahm F, Hamelmann S, Ratliff M et al (2025) Analysis of anatomical location, mitoses, and Ki-67 in 2608 meningiomas. J Neuropathol Exp Neurol. 10.1093/jnen/nlaf131 10.1093/jnen/nlaf131 41267161

[20] Mirian C, Skyrman S, Bartek JJ, Jensen LR, Kihlström L, Förander P et al (2020) The Ki-67 proliferation index as a marker of time to recurrence in intracranial meningioma. Neurosurg 87:1289. 10.1093/neuros/nyaa226 10.1093/neuros/nyaa226 32614441

[21] Backer-Grøndahl T, Moen BH, Sundstrøm SH, Torp SH (2014) Histopathology and prognosis in human meningiomas. APMIS 122:856–866. 10.1111/apm.12248 24698127 10.1111/apm.12248

[22] Rogers CL, Perry A, Pugh S, Vogelbaum MA, Brachman D, McMillan W et al (2016) Pathology concordance levels for meningioma classification and grading in NRG oncology RTOG trial 0539. Neuro-oncol 18:565–574. 10.1093/neuonc/nov247 26493095 10.1093/neuonc/nov247 PMC4799683

[23] Abry E, Thomassen IØ, Salvesen ØO, Torp SH (2010) The significance of Ki-67/MIB-1 labeling index in human meningiomas: a literature study. Pathol Res Pract 206:810–815. 10.1016/j.prp.2010.09.002 20951502 10.1016/j.prp.2010.09.002

[24] Polley M-YC, Leung SCY, Gao D, Mastropasqua MG, Zabaglo LA, Bartlett JMS, et al (2015) An international study to increase concordance in Ki67 scoring. Mod Pathol. Nature Publishing Group 28:778–86. 10.1038/modpathol.2015.38 10.1038/modpathol.2015.38 25698062

[25] Bankhead P, Loughrey MB, Fernández JA, Dombrowski Y, McArt DG, Dunne PD et al (2017) QuPath: open source software for digital pathology image analysis. Sci Rep 7:16878. 10.1038/s41598-017-17204-5 29203879 10.1038/s41598-017-17204-5 PMC5715110

[26] Thirunavu V, Drumm M, McCord M, Steffens A, Youngblood MW, Nandoliya KR et al (2024) Optimizing the use of Ki-67 proliferative index as a prognostic biomarker in meningiomas using digital analysis. J Neurosurg 141:1644–1654. 10.3171/2024.4.JNS232857 38968615 10.3171/2024.4.JNS232857 PMC12192485

[27] Nowak-Choi K, Palmer JD, Casey J, Chitale A, Kalchman I, Buss E et al (2021) Resected WHO grade I meningioma and predictors of local control. J Neurooncol 152:145–151. 10.1007/s11060-020-03688-1 33420897 10.1007/s11060-020-03688-1

[28] Kanwal N, Perez-Bueno F, Schmidt A, Engan K, Molina R (2022) The devil is in the details: whole slide image acquisition and processing for artifacts detection, color variation, and data augmentation: a review. IEEE Access 10:58821–58844. 10.1109/ACCESS.2022.3176091

[29] Kahaki S, Webber AR, Zamzmi G, Subbaswamy A, Deshpande R, Badano A (2025) HistoART: Histopathology artifact detection and reporting tool [Internet]. arXiv; 10.48550/arXiv.2507.00044 [cited 2 Dec 2025]

[30] Simpson D (1957) The recurrence of intracranial meningiomas after surgical treatment. J Neurol Neurosurg Psychiatry 20:22 13406590 10.1136/jnnp.20.1.22 PMC497230

[31] Clark VE, Erson-Omay EZ, Serin A, Yin J, Cotney J, Ozduman K et al (2013) Genomic analysis of non-NF2 meningiomas reveals mutations in TRAF7, KLF4, AKT1, and SMO. Sci 339:1077–1080. 10.1126/science.1233009 10.1126/science.1233009 PMC4808587 23348505

[32] Mansouri A, Klironomos G, Taslimi S, Kilian A, Gentili F, Khan OH et al (2016) Surgically resected skull base meningiomas demonstrate a divergent postoperative recurrence pattern compared with non-skull base meningiomas. J Neurosurg 125:431–440. 10.3171/2015.7.JNS15546 26722844 10.3171/2015.7.JNS15546

[33] Youngblood MW, Duran D, Montejo JD, Li C, Omay SB, Özduman K et al (2020) Correlations between genomic subgroup and clinical features in a cohort of more than 3000 meningiomas. J Neurosurg 133:1345–1354. 10.3171/2019.8.JNS191266 31653806 10.3171/2019.8.JNS191266

[34] Dosovitskiy A, Beyer L, Kolesnikov A, Weissenborn D, Zhai X, Unterthiner T, et al (2021) An image is worth 16x16 words: transformers for image recognition at scale [Internet]. arXiv; 10.48550/arXiv.2010.11929 [cited 2 Dec 2025]

[35] Chen RJ, Ding T, Lu MY, Williamson DFK, Jaume G, Song AH et al (2024) Towards a general-purpose foundation model for computational pathology. Nat Med 30:850–862. 10.1038/s41591-024-02857-3 38504018 10.1038/s41591-024-02857-3 PMC11403354

[36] Daenekas B, Pérez E, Boniolo F, Stefan S, Benfatto S, Sill M et al (2024) Conumee 2.0: enhanced copy-number variation analysis from DNA methylation arrays for humans and mice. Bioinforma Oxf Engl 40(2):btae029 10.1093/bioinformatics/btae029 10.1093/bioinformatics/btae029 PMC10868300 38244574

[37] Hielscher T, Sill M, Sievers P, Stichel D, Brandner S, Jones DTW et al (2022) Clinical implementation of integrated molecular‐morphologic risk prediction for meningioma. Brain Pathol 33:e13132. 10.1111/bpa.13132 36377252 10.1111/bpa.13132 PMC10154374

[38] Therneau TM, Grambsch PM (2000) The cox model. In: Therneau TM, Grambsch PM (eds) Model surviv data extending cox model. Springer New York, New York, NY, pp 39–77. 10.1007/978-1-4757-3294-8_3

[39] Akaike H (1998) A new look at the statistical model identification. In: Parzen E, Tanabe K, Kitagawa G (eds) Sel pap Hirotugu Akaike. Springer, New York, NY, pp 215–222. 10.1007/978-1-4612-1694-0_16

[40] Heagerty PJ, Lumley T, Pepe MS (2000) Time-dependent ROC curves for censored survival data and a diagnostic marker. Biometrics 56:337–344. 10.1111/j.0006-341x.2000.00337.x 10877287 10.1111/j.0006-341x.2000.00337.x

[41] Youden WJ (1950) Index for rating diagnostic tests. Cancer 3:32–5. 10.1002/1097-0142(1950)3:1<32::aid-cncr2820030106>3.0.co;2-3 10.1002/1097-0142(1950)3:1<32::aid-cncr2820030106>3.0.co;2-3 15405679

[42] Koo TK, Li MY (2016) A guideline of selecting and reporting intraclass correlation coefficients for reliability research. J Chiropr Med 15:155–163. 10.1016/j.jcm.2016.02.012 27330520 10.1016/j.jcm.2016.02.012 PMC4913118

[43] Guo X, Patel RV, Lederer JA, Meredith DM, Bi WL (2025) Ki-67 in meningioma: distribution and implications. J Neurosurg 143:1325–1335. 10.3171/2025.4.JNS25438 40712166 10.3171/2025.4.JNS25438

[44] Ugwuowo U, Faust B, Tang H, DiStasio M (2025) Adequate capture of spatial heterogeneity of Ki-67 proliferative index in meningiomas requires multiple tissue sections. J Neuropathol Exp Neurol 84:1067–1070. 10.1093/jnen/nlaf043 40257502 10.1093/jnen/nlaf043 PMC12531482

[45] Pham DT, Skaland I, Winther TL, Salvesen Ø, Torp SH (2020) Correlation between digital and manual determinations of Ki-67/MIB-1 proliferative indices in human meningiomas. Int J Surg Pathol 28:273–279. 10.1177/1066896919889148 31771372 10.1177/1066896919889148

[46] Mizrachi M, Hartley B, Saleem S, Hintz E, Ziemba Y, Li J et al (2024) Ki-67 index as a predictive marker of meningioma recurrence following surgical resection. J Clin Neurosci 124:15–19. 10.1016/j.jocn.2024.04.015 38631196 10.1016/j.jocn.2024.04.015

[47] Wagle PR, Loeschner D, Rosahl S, Brodhun M, Gerlach R (2024) A comprehensive correlation of the KI-67 proliferation index to patient’s, imaging and tumor features and its value in predicting long-term course of patients with newly diagnosed intracranial meningiomas. Neurosurg Rev 47:241. 10.1007/s10143-024-02485-y 38806958 10.1007/s10143-024-02485-y

[48] Gauchotte G, Bédel C, Lardenois E, Hergalant S, Cuglietta L, Pflaum R et al (2023) A high MCM6 proliferative index in atypical meningioma is associated with shorter progression free and overall survivals. Cancers (Basel) 15:535. 10.3390/cancers15020535 36672484 10.3390/cancers15020535 PMC9857276

[49] van den Berg EJ, Duarte R, Dickens C, Joffe M, Mohanlal R (2021) KI67 immunohistochemistry quantification in breast carcinoma: a comparison of visual estimation, counting and Immunoratio©. Appl Immunohistochem Mol Morphol 29:105–111. 10.1097/PAI.0000000000000864 32590453 10.1097/PAI.0000000000000864 PMC7755692

[50] Guresci S, Aydogdu OB, Secen AE, Uzel B (2025) Assessing the predictive value of Ki-67 and progesterone receptor algorithms for recurrence and disease-free survival in meningiomas. Ann Diagn Pathol 75:152441. 10.1016/j.anndiagpath.2025.152441 39842300 10.1016/j.anndiagpath.2025.152441

[51] Bankhead P (2022) Developing image analysis methods for digital pathology. J Pathol 257:391–402. 10.1002/path.5921 35481680 10.1002/path.5921 PMC9324951

[52] Rewcastle E, Skaland I, Gudlaugsson E, Fykse SK, Baak JPA, Janssen EAM (2024) The Ki67 dilemma: investigating prognostic cut-offs and reproducibility for automated Ki67 scoring in breast cancer. Breast Cancer Res Treat 207:1–12. 10.1007/s10549-024-07352-4 38797793 10.1007/s10549-024-07352-4 PMC11231004

[53] Swiderska Z, Korzynska A, Markiewicz T, Lorent M, Zak J, Wesolowska A et al (2015) Comparison of the manual, semiautomatic, and automatic selection and leveling of hot spots in whole slide images for Ki-67 quantification in meningiomas. Anal Cell Pathol Amst 2015:498746. 10.1155/2015/498746 26240787 10.1155/2015/498746 PMC4512563

[54] Ersay H, Hatipoglu HG, Guresci S (1987) ADC values compared to tumor grade and Ki-67 proliferation index detected by a digital image analysis program in meningiomas. Acta Radiol 66:1263–1270. 10.1177/02841851251365512 10.1177/02841851251365512 PMC12669402 40820337

[55] Grala B, Markiewicz T, Kozłowski W, Osowski S, Słodkowska J, Papierz W (2009) New automated image analysis method for the assessment of Ki-67 labeling index in meningiomas. Folia Histochem Cytobiol 47:587–592. 10.2478/v10042-008-0098-0 20430724 10.2478/v10042-008-0098-0

[56] Shukla IY, Ebada A, Bever N, Traylor JI, Wan B, Shah D et al (2025) Prognostic value of MIB-1 index in meningioma: a retrospective cohort study to establish an optimal cutoff for recurrence and survival. J Neurooncol 174:483–492. 10.1007/s11060-025-05057-2 40353934 10.1007/s11060-025-05057-2

[57] Bruna J, Brell M, Ferrer I, Gimenez-Bonafe P, Tortosa A (2007) Ki-67 proliferative index predicts clinical outcome in patients with atypical or anaplastic meningioma. Neuropathol 27:114–120. 10.1111/j.1440-1789.2007.00750.x 10.1111/j.1440-1789.2007.00750.x 17494511

[58] Wang F, Xu D, Liu Y, Lin Y, Wei Q, Gao Q et al (2019) Risk factors associated with postoperative recurrence in atypical intracranial meningioma: analysis of 263 cases at a single neurosurgical centre. Acta Neurochir (Wien) 161:2563–2570. 10.1007/s00701-019-04073-2 31641861 10.1007/s00701-019-04073-2

[59] Olar A, Wani KM, Wilson CD, Zadeh G, DeMonte F, Jones DT et al (2017) Global epigenetic profiling identifies methylation subgroups associated with recurrence-free survival in meningioma. Acta Neuropathol 133:431–444. 10.1007/s00401-017-1678-x 28130639 10.1007/s00401-017-1678-x PMC5600514

[60] Singh J, Sharma R, Shukla N, Narwal P, Katiyar A, Mahajan S et al (2023) DNA methylation profiling of meningiomas highlights clinically distinct molecular subgroups. J Neurooncol 161:339–356. 10.1007/s11060-022-04220-3 36564673 10.1007/s11060-022-04220-3

[61] Duckett D, Santana-Santos L, McCord M, Smith V, Lopes MBS, Youngblood MW et al (2025) Development, validation, and utility of a clinically applicable methylation classifier for recurrence risk prediction in meningiomas. Acta Neuropathol Commun 13:259. 10.1186/s40478-025-02184-9 41466325 10.1186/s40478-025-02184-9 PMC12750935

[62] Hergalant S, Saurel C, Divoux M, Rech F, Pouget C, Godfraind C et al (2022) Correlation between DNA methylation and cell proliferation identifies new candidate predictive markers in meningioma. Cancers 14:6227. 10.3390/cancers14246227 36551712 10.3390/cancers14246227 PMC9776514

[63] Altman DG, Royston P (2006) The cost of dichotomising continuous variables. BMJ 332:1080. 10.1136/bmj.332.7549.1080 16675816 10.1136/bmj.332.7549.1080 PMC1458573

[64] Hua S, Yan F, Shen T, Ma L, Zhang X (2024) PathoDuet: foundation models for pathological slide analysis of H&E and IHC stains [Internet]. arXiv; 10.48550/arXiv.2312.09894 [cited 8 Mar 2026] 10.1016/j.media.2024.103289 39106763

[65] MahmoodLab. UNI2-h: pathology foundation model [Internet]. [cited 8 Mar 2026 ]. https://huggingface.co/MahmoodLab/UNI2-h. Accessed 8 Mar 2026

[66] Sehring J, Dohmen H, Selignow C, Schmid K, Grau S, Stein M et al (2023) Leveraging attention-based convolutional neural networks for meningioma classification in computational histopathology. Cancers 15:5190. 10.3390/cancers15215190 37958364 10.3390/cancers15215190 PMC10647687

[67] Ayad MA, McCortney K, Congivaram HTS, Hjerthen MG, Steffens A, Zhang H, et al (2026) Morphological set enrichment enables interpretable prognostication and molecular profiling of meningiomas [Internet]. medRxiv; p. 2026.02.23.26346491. 10.64898/2026.02.23.26346491 [cited 12 Apr 2026]

[68] Hoang D-T, Shulman ED, Turakulov R, Abdullaev Z, Singh O, Campagnolo EM et al (2024) Prediction of DNA methylation-based tumor types from histopathology in central nervous system tumors with deep learning. Nat Med 30:1952–1961. 10.1038/s41591-024-02995-8 38760587 10.1038/s41591-024-02995-8

[69] Hoang D-T, Shulman ED, Dhruba SR, Nair NU, Barman RK, Lalchungnunga H, et al (2025) Path2Omics: enhanced transcriptomic and methylation prediction accuracy from tumor histopathology. bioRxiv. ;2025.02.26.640189. 10.1101/2025.02.26.640189 10.1158/0008-5472.CAN-25-4350 PMC12704547 41166699

