# Supplementary material for: Time-dependent prognostic value of automated Ki67 assessment and its integration with molecular risk profiling in WHO grade 2 meningioma
Source: Acta Neuropathol Commun. 2026 Jul 27;14:157. doi: 10.1186/s40478-026-02387-8 (PMC13411134; doi:10.1186/s40478-026-02387-8)
Supplement: Supplementary file 1 — Additional file1 (DOCX 229 KB) [file 40478_2026_2387_MOESM1_ESM.docx]

Supplementary Material

# Supplementary Figure S1. AIC grid-search for the optimal time split


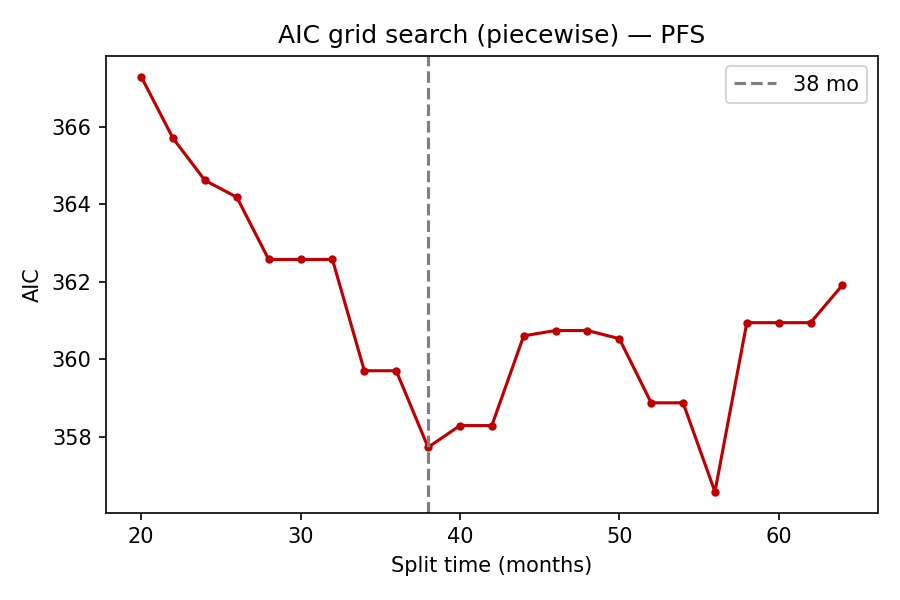


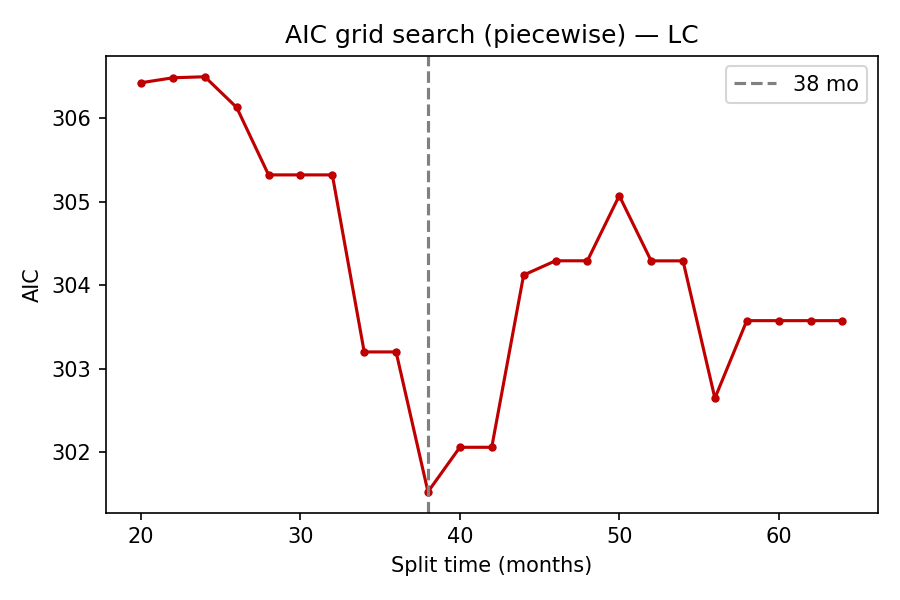


*Supplementary Figure S1. Akaike Information Criterion (AIC) of the piecewise-constant Cox model as a function of the time split for PFS (left) and LC (right). The dashed line marks the 38-month split selected as the convergent optimum across endpoints (PFS AIC 357.72; LC AIC 301.53). For PFS, two near-equivalent local optima are apparent at 38 and 56 months (ΔAIC = 1.2), whereas for LC 38 months is the distinct optimum; 38 months was selected as the split convergent across both endpoints.*

# Supplementary Figure S2. Time-dependent ROC analysis at 38 months


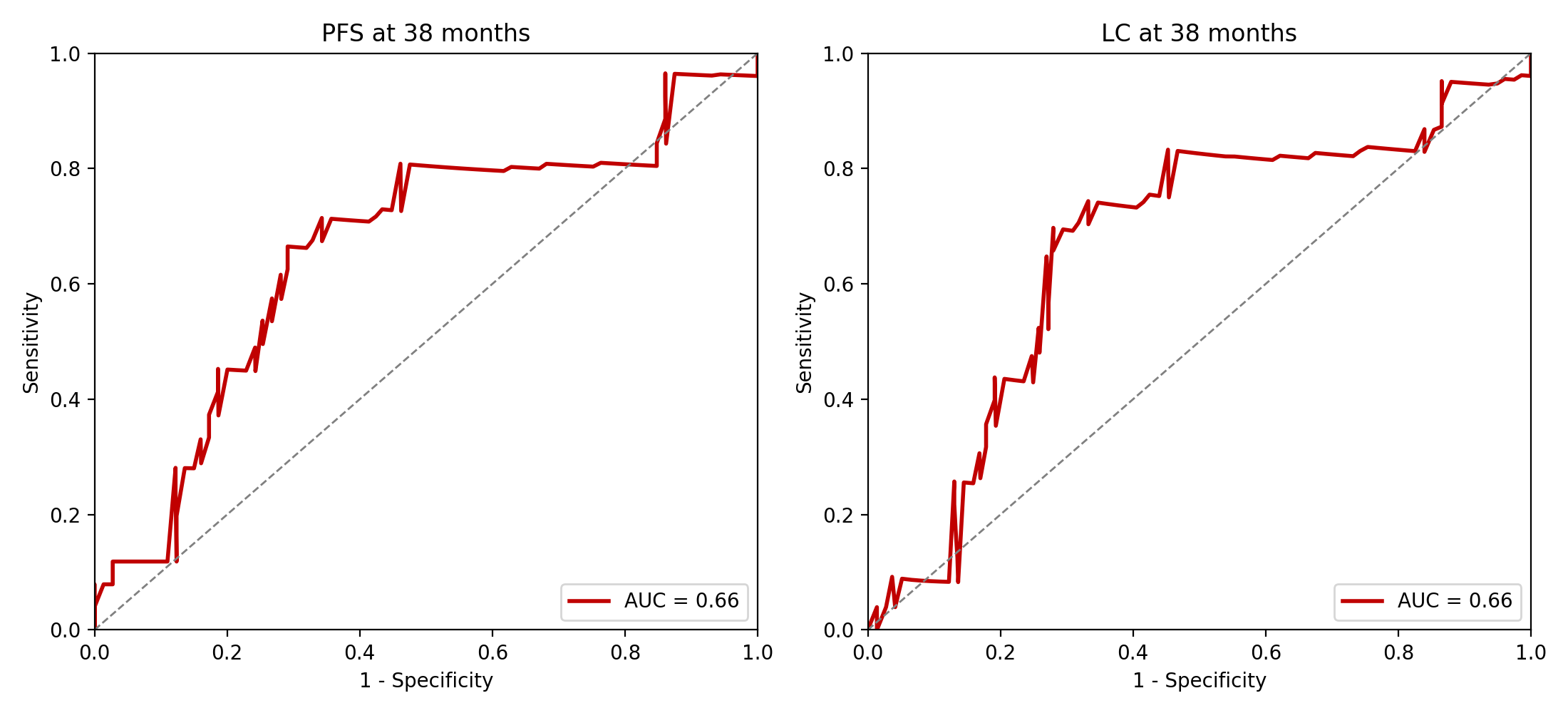


*Supplementary Figure S2. Time-dependent receiver operating characteristic (ROC) curves for the automated Ki67 index at 38 months for progression-free survival (PFS, left) and local control (LC, right). The area under the curve (AUC) was 0.66 for both endpoints. The Youden-derived optimal cut-off was 3.62% (PFS: sensitivity 66.5%, specificity 70.9%; LC: sensitivity 69.7%, specificity 72.0%).* Curves are empirical (step-function) time-dependent ROC estimates (Heagerty Kaplan–Meier method); the stepwise appearance reflects the discrete event times.

# Supplementary Table S1. Automated versus visual Ki67: early-phase prognostic comparison

| **Endpoint** | **Measure** | **Continuous HR (per % point)** | **p** | **Binary HR (high vs. low Ki67)** | **p** | **n** |
| --- | --- | --- | --- | --- | --- | --- |
| PFS | Automated (artifact-adjusted) | 1.24 | 0.004 | 3.43 at 3.62% | 0.004 | 98 |
| PFS | Visual – maximum | 1.07 | 0.041 | 1.87 at 8% | 0.150 | 96 |
| PFS | Visual – midpoint | 1.08 | 0.022 | 2.07 at 7.5% | 0.094 | 96 |
| PFS | Visual – minimum | 1.09 | 0.036 | 2.07 at 5% | 0.094 | 96 |
| LC | Automated (artifact-adjusted) | 1.18 | 0.060 | 3.91 at 3.62% | 0.003 | 98 |
| LC | Visual – maximum | 1.05 | 0.249 | 2.03 at 5% | 0.117 | 96 |
| LC | Visual – midpoint | 1.05 | 0.178 | 2.03 at 5% | 0.117 | 96 |
| LC | Visual – minimum | 1.07 | 0.121 | 1.90 at 5% | 0.141 | 96 |

*Supplementary Table S1. Early-phase (≤38 months) piecewise Cox models comparing the automated index with the retrospective visual indices (two purely qualitative reports coded “n/a” excluded; visual n=96). Visual indices were tested as the minimum, midpoint, and maximum of each reported range, each dichotomized at its own ROC-derived cut-off. The automated measure was the stronger and more consistent predictor, reaching significance for both endpoints on dichotomization; the visual index showed at most a weak continuous PFS association and did not reach significance when dichotomized. Over the full follow-up neither index was individually significant, consistent with the time-dependent nature of the effect.*

# Supplementary Table S2. Automated hotspot versus global Ki67

| **Quantification method** | **Early HR LC (95% CI)** | **p** | **Early HR PFS (95% CI)** | **p** | **AUC @38 (LC/PFS)** | **C-index (LC/PFS)** |
| --- | --- | --- | --- | --- | --- | --- |
| Global (artifact-adjusted) | 3.91 (1.60–9.52) | 0.003 | 3.43 (1.47–8.03) | 0.004 | 0.66 / 0.66 | 0.60 / 0.61 |
| Automated hotspot (highest-Ki67 tile) | 4.95 (1.67–14.74) | 0.004 | 5.44 (1.84–16.08) | 0.002 | 0.66 / 0.70 | 0.64 / 0.67 |

*Supplementary Table S2. Automated hotspot Ki67: each whole-slide image was sampled with 75 tiles of ≈0.25 mm² (15 tiles in each of five sampling runs), the Ki67 index was computed per tile, and the hotspot was defined as the highest per-tile Ki67 index (tiles with ≥100 detected cells; n=95 with available tile-level data). As this samples rather than exhaustively searches the section, it approximates the true hotspot. Each index was dichotomized at its own ROC-derived early-phase cut-off (global 3.62%; hotspot 10.48%). The hotspot index was ~3-fold higher than the global mean and closely matched the routine visual median (10.1% vs 10.0%; ρ=0.63 with the visual indices). Hotspot and global quantification correlated (ρ=0.74) and showed comparable early-phase prognostic discrimination, with neither clearly superior.*

# Supplementary Table S3. Sensitivity analysis restricted to primary resections

| **Endpoint** | **Cohort** | **n** | **HR (Ki67 >3.62%)** | **95% CI** | **p** |
| --- | --- | --- | --- | --- | --- |
| LC | Full cohort | 98 | 3.91 | 1.60–9.52 | 0.003 |
| LC | Primary only | 95 | 3.77 | 1.53–9.26 | 0.004 |
| PFS | Full cohort | 98 | 3.43 | 1.47–8.03 | 0.004 |
| PFS | Primary only | 95 | 3.30 | 1.40–7.80 | 0.006 |

*Early-phase (≤38 months) piecewise Cox models. Exclusion of the three recurrent-tumor surgeries left the estimates essentially unchanged.*

# Supplementary Table S4. Clinical and molecular characteristics of early vs. late/no recurrence in the high-Ki67 subgroup (>3.62%)

| **Variable** | **Early event (≤38 mo, n=16)** | **Remainder (n=22)** | **p** |
| --- | --- | --- | --- |
| Male sex | 11 (69%) | 10 (45%) | 0.197 |
| Subtotal resection | 4 (25%) | 2 (9%) | 0.217 |
| Integrated risk group (low/int/high) | 0/12/4 | 9/9/4 | 0.013 |
| 1p loss | 16 (100%) | 11 (50%) | 0.001 |
| 6q loss | 8 (50%) | 2 (9%) | 0.008 |
| 14q loss | 9 (56%) | 7 (32%) | 0.188 |
| Adjuvant radiotherapy | 3 (19%) | 6 (27%) | 0.706 |
| Age, median (years) | 64.0 | 60.4 | 0.668 |
| Ki67, median (%) | 4.49 | 4.75 | 0.668 |

*Within the high-Ki67 subgroup (>3.62%, n=38), early recurrers did not differ in Ki67 level but carried a significantly higher molecular burden (1p/6q loss, integrated risk group). Fisher’s exact (categorical) and Mann-Whitney U (continuous).*

# Supplementary Table S5. Benchmark of the artifact-adjusted pipeline against uncorrected quantification

| **Quantification / endpoint** | **AUC @38 mo** | **Ki67 cut-off** | **HR (95% CI)** | **p** | **C-index** |
| --- | --- | --- | --- | --- | --- |
| Global (unadjusted) – PFS | 0.67 | 4.06% | 3.38 (1.48–7.74) | 0.004 | 0.62 |
| Global (unadjusted) – LC | 0.67 | 3.02% | 3.89 (1.44–10.48) | 0.007 | 0.62 |
| Artifact-adjusted – PFS | 0.66 | 3.62% | 3.43 (1.47–8.03) | 0.004 | 0.61 |
| Artifact-adjusted – LC | 0.66 | 3.62% | 3.91 (1.60–9.52) | 0.003 | 0.60 |

*Supplementary Table S5. Fair benchmark of the artifact-adjusted (HistoART) versus the uncorrected global quantification (QuPath without HistoART artifact exclusion), using cutoff-independent metrics (time-dependent AUC at 38 months and the continuous concordance index) and each index evaluated at its own ROC-derived optimal cut-off (global: LC 3.02%, PFS 4.06%; artifact-adjusted both: 3.62%). The two quantifications were prognostically equivalent; artifact removal safeguards measurement validity rather than improving prognostic discrimination in this cohort of predominantly large, good-quality sections.*

# Supplementary Table S6. Number of patients at risk over time for local control (LC), stratified by automated Ki67 proliferation index

| **Group** | **0** | **12** | **24** | **38** | **54** | **72** | **96** | **120** |
| --- | --- | --- | --- | --- | --- | --- | --- | --- |
| Ki67 >3.62% (high, n=38) | 38 | 35 | 27 | 16 | 14 | 8 | 5 | 3 |
| Ki67 ≤3.62% (low, n=60) | 60 | 57 | 43 | 41 | 23 | 17 | 7 | 4 |

*Number at risk (months; local control timeline). The high-Ki67 group is substantially depleted at later follow-up, limiting the power to characterize the late phase.*
